# Exploration of the roles of SSR2 in hepatocellular carcinogenesis based on single-cell transcriptomics and spatial transcriptomics

**DOI:** 10.1007/s12672-026-05017-w

**Published:** 2026-04-14

**Authors:** Siyuan Liu, Xuyang Wang, Donghao Cheng, Kaipeng Hu, Xihu Qin

**Affiliations:** 1 Department of Hepato-Biliary-Pancreatic Surgery, The Second People’s Hospital of Changzhou, The Third Affiliated Hospital of Nanjing Medical University, Changzhou, China; 2 The First Clinical Medical College of Anhui Medical University, Hefei, 230032 China; 3 The First Affiliated Hospital of Anhui Medical University, Hefei, China

**Keywords:** Hepatocellular carcinoma, Natural killer cells, SSR2, Cytoplasmic translation, MIF, Single-cell transcriptome, Spatial transcriptome, Machine learning, Tumor immune evasion

## Abstract

**Background:**

Hepatocellular carcinoma (HCC) is one of the most common types of cancer globally. However, HCC features poor prognosis due to complex pathogenesis and limitations of therapeutic approaches.

**Method:**

To improve the prognosis of HCC patients, we analyzed single-cell transcriptome and spatial transcriptome on liver tissues from HCC patients. We applied Non-negative Matrix Factorization (NMF) method on spatial transcriptome and found a critical pathway associated with HCC through GO over-representation analysis. According to the pathway activity score, the cells in single-cell transcriptome were divided into three groups. Then we conducted hdWGCNA and selected HCC-related co-expressed gene modules which showed significant intergroup differences. Integrative machine learning algorithms on bulk transcriptome were employed for core genes selection. We respectively evaluated their AUC values as independent diagnostic markers and retained a core gene. We conducted a survival analysis using this gene. Next, we performed immune infiltration analysis, tumor microenvironmental analysis and drug sensitivity analysis. Finally, pseudotime analysis, differentiation potential analysis and cell–cell communication analysis were applied to decipher the mechanisms underlying tumor initiation and progression.

**Results:**

Single-cell transcriptome analysis revealed the high heterogeneity of cells in patients with HCC. The GO over-representation analysis based on NMF indicated that cytoplasmic translation path was enriched in most NMF components. The SSR2 gene passed the integrative machine learning algorithms and possessed excellent diagnosis (AUC > 0.8) and prognostic abilities (K-M curve, P = 0.007). Further analysis identified SSR2 as the top-ranked gene by average expression level in NK cells compared to all other immune cell clusters. Its expression showed a consistent downward trend during NK cell maturation.NK cells with high expression of SSR2 exhibited stronger cell–cell communication than those with low expression of SSR2. NK cells mainly communicated with other cells through MIF-mediated signaling pathway.

**Conclusion:**

In hepatocellular carcinoma (HCC), the high expression of the SSR2 gene in NK cells is accompanied by enhanced cytoplasmic translation pathway, which promotes MIF secretion and activates its downstream signaling pathways, thereby driving the disruption of the tumor immune microenvironment and the progression of the disease.

**Supplementary Information:**

The online version contains supplementary material available at 10.1007/s12672-026-05017-w.

## Introduction

Hepatocellular carcinoma (HCC) is among the most common and lethal cancers globally [1], especially in relation to chronic liver conditions such hepatitis B or C infections and liver cirrhosis [2]. Despite the significant enhancement of outcomes for specific patient subsets through surgical resection, locoregional therapies, and targeted agents like tyrosine kinase inhibitors, HCC continues to exhibit a high recurrence rate and challenges in early diagnosis, leading to consistently low overall 5-year survival rates [3]. This adverse outcome is primarily due to the intricate nature of the tumor immune microenvironment (TIME) and the various mechanisms of immune evasion [4–6].

Natural killer (NK) cells, as an essential element of the innate immune system, are pivotal in inhibiting tumor advancement [7]. In HCC, NK-cell subsets demonstrate significant heterogeneity. Numerous investigations have revealed both highly cytotoxic, activated NK cells and NK cells in a ‘exhausted’ condition, potentially linked to regulatory impacts from the tumor microenvironment (TME) [5]. In the HCC microenvironment, NK cells demonstrate significant dysfunction. Research indicates that these NK cells have decreased release of cytokines, including IFN-γ and TNF-α, as well as lower cytotoxic action [8, 9]. The mechanisms involved are complex: tumor cells may evade recognition by NK cells through the downregulation of ligands for activating receptors (e.g., NKG2D) or by secreting soluble decoy forms like sMICA; concurrently, the upregulation of inhibitory receptors on NK cells (e.g., NKG2A, KIR) diminishes their cytolytic efficacy [9]. Furthermore, immunosuppressive cells in the tumor microenvironment—such as myeloid-derived suppressor cells (MDSC), tumor-associated macrophages (TAM), and regulatory T cells—alongside stromal elements like cancer-associated fibroblasts (CAF), secrete mediators including TGF-β, IL-10, PGE₂, and IDO, all of which inhibit NK-cell activity and cytotoxicity [10].

As single-cell RNA sequencing (scRNA-seq) and extensive bulk RNA-seq datasets proliferate, researchers are progressively equipped to analyze cellular heterogeneity and intercellular interactions within the immunological microenvironment of HCC [11]. Multiple researches have utilized these datasets to develop cell–cell communication networks, uncover critical ligand–receptor combinations, and employ machine-learning techniques—such as feature selection and clustering analyses—to find gene signatures that may affect immune-cell performance [12]. Emerging evidence indicates that cytoplasmic translation is a key regulator of immune-cell effector function. In natural killer (NK) cells, efficient protein synthesis is required for the rapid production of cytotoxic molecules and cytokines, including perforin, granzymes, and IFN-γ [13–15]. Moreover, translational dysregulation driven by tumor-associated stress and immunosuppressive signals—such as TGF-β–mediated inhibition of mTOR signaling—has been implicated in NK-cell dysfunction and exhaustion within the tumor microenvironment [16]. However, the translational regulatory features of NK-cell subsets in hepatocellular carcinoma remain insufficiently characterized, particularly at the single-cell level. This study builds on current approaches by integrating single-cell transcriptome data from HCC to thoroughly define NK-cell subset architecture and their communication networks with other cell populations in the tumor microenvironment. We subsequently integrated single-cell data with bulk RNA-seq profiles and employed machine-learning techniques to discern key genes associated with NK-cell functional states, specifically those related to cytoplasmic translation.

## Materials and methods

### Data source

HCC scRNA-seq was obtained from GEO database (Accession ID: GSE149614) [17], including 21 samples from 10 HCC patients’ different tissues. We chose 10 Single cells samples in tumor tissues of HCC patients to study on the cellular heterogeneity of tumor tissues in HCC patients.

**Fig. 1:**
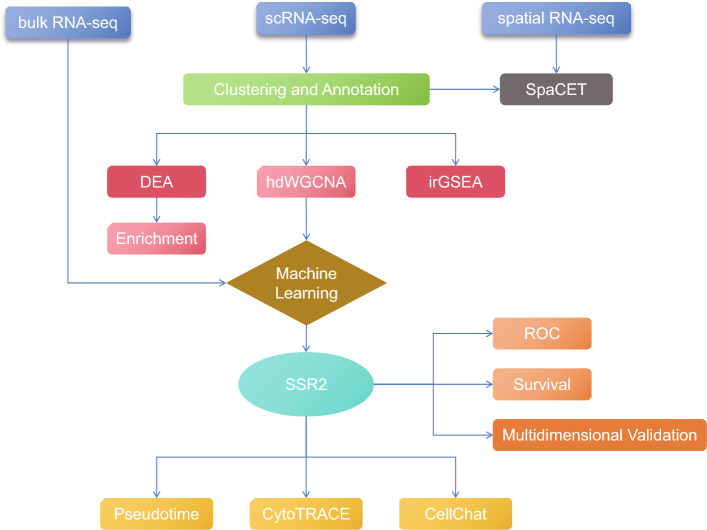
Workflow diagram for the analysis

In order to further study the spatial distribution and interactions of single cells in HCC patients, we downloaded the spatial RNA-seq from GEO database [Accession ID: GSE203612 (GSM6177612)] [18]. In TCGA, we obtained 374 liver cancer samples and 50 adjacent normal samples from 371 liver cancer patients (some patients were sampled multiple times) to screen for core genes. In addition, GSE76427 served as a validation cohort [19]. The overall workflow diagram is shown in Fig. 1.

### Single-cell transcriptome data processing

R (version 4.5.1) was adopted to process the data, conduct subsequent analysis and perform visualization. For single-cell transcriptome data, the Seurat package (version 5.3.1) was used to create a Seurat object, retaining only the genes expressed in 5 cells and only the cells that express at least 300 genes [20]. To eliminate invalid data and prevent low-quality cells from interfering with the subsequent analysis, we selected cells with the mitochondrial gene ratio of less than 25%, the ribosomal gene ratio of more than 3%, and the hemoglobin gene ratio of less than 1%. Then we used “LogNormalize” method to perform normalization, followed by data scaling. FindVariableFeatures function with default parameters was applied to select highly variable genes. After principal component analysis (PCA), we performed batch effect correction and integration through Harmony package. We constructed the nearest neighbor graph based on the first 15 principal components, performed clustering with a resolution of 0.8, and finally visualized it using UMAP. Cell type markers were collected from previously published literature to create a reference list. Clusters were annotated according to observe known cell type markers’ expression.

### Differential expression and over-representation analysis

Differentially expressed genes (DEGs) were identified by comparing gene expression profiles across clusters using FindAllMarkers function with min expression fraction = 0.25 and LogFC threshold = 0.25. To explore the biological functions and signal pathways, Gene Ontology (GO) over-representation analysis (ORA) for Biological Process (BP) was performed on cluster-specific DEGs using enrichCluster function.

### Gene set enrichment analysis

Gene set enrichment analysis (GSEA) was employed to assess the enrichment of hallmark pathways from MSigDB Hallmark gene sets using the irGSEA package. Enrichment score was calculated with the following methods: AUCell (gene-ranking AUC), UCell (Mann–Whitney U), singscore (rank permutation), ssgsea (weighted rank), JASMINE (network-based), and viper (regulon inference).Consensus hallmark pathways were derived by intersecting significantly enriched gene sets across all six methodologies.

### Spatial transcriptome data processing and analysis

The spatial transcriptome data was processed using Seurat package (version 5.3.1), including data loading, normalizing and finding highly variable genes. Non-negative Matrix Factorization (NMF) was implemented using RunNMF function with default parameters. To evaluate the rationality of the factorization rank, the inflection point position was selected as the best rank (k = 6) through RankPlot function. The top 30 genes for every NMF component extracted to perform GO over-representation analysis. The pathway gene set that was significantly enriched in the largest number of NMF components was selected as the key gene set (cytoplasmic translation).

### Cell scoring

Cell scoring was performed using the UCell package on single-cell transcriptome data according to the key gene set from spatial transcriptome data. Cells were stratified into three groups based on the tertiles of their UCell scores: “High_CT” (upper tertile, top 1/3), “Low_CT” (lower tertile, bottom 1/3), and “Medium” (middle 1/3).

### High-dimensional weighted correlation network analysis (hdWGCNA)

Co-expressed gene modules were identified using high-dimensional weighted correlation network analysis (hdWGCNA) [21]. After filtering for genes expressed in ≥ 5% of cells, we constructed metacells (25 nearest neighbors; max 10 shared cells) grouped by cellular activity states (“High_CT”, “Low_CT”, “Medium”).Then we focused on the “High_CT” group, extracted the expression matrix of the metacells in this group, and used it as the input for co-expression analysis.A signed gene co-expression network was built with soft-thresholding (power = 8) to achieve scale-free topology. Hierarchical clustering identified initial gene modules, which were merged (dendrogram cut height = 0.2) into distinct co-expression modules (minimum size = 50 genes). Module eigengenes were computed as the first principal component of each cluster, and hub genes were defined as the top 10 genes by intramodular connectivity (kME). Two biologically significant modules (black and green) were prioritized for downstream functional characterization according to expression differences of gene modules among cellular activity states (“High_CT”, “Low_CT”, “Medium”).

### Bulk transcriptome data processing

The gene expression matrices from TCGA (RNA-seq) and GEO (microarray) cohorts were integrated. The samples with complete clinical annotations was retained. TCGA RNA-seq data and GEO microarray data provided had undergone normalization. Log2 transformation was applied to the GEO microarray data, as the TCGA RNA-seq data were already log2-transformed. To address technical variations, we performed cross-platform batch correction using ComBat (sva package) with "dataset" as the batch covariate. Subsequently, we extracted the gene expression levels of the significantly identified modules obtained through WGCNA (black and green) from the processed expression matrix and integrated the clinical information (“Tumor” or “Normal”) for machine learning.

### Integrative machine learning for core genes selection

Seven machine learning algorithms (LASSO, SVM-RFE, Random Forest, Boruta, Decision Tree, XGBoost, GBM) were employed, To ensure the robustness of feature selection, the entire TCGA bulk transcriptome dataset was utilized as the discovery (training) set, while the GEO dataset was employed as an independent external testing set for validation [22–28]. Feature selection was performed using the Tumor vs. Normal labels as the classification. LASSO regression identified features via tenfold cross-validated L1 regularization. SVM-RFE performed recursive feature elimination with linear kernels. Random Forest leveraged 500-tree ensembles with Gini importance ranking. Boruta used shadow feature methodology with 300 iterations at α = 0.05 significance. Decision Trees employed maximal-depth partitioning (cp = 10–10). XGBoost implemented gradient boosting with 50 iterations, learning rate η = 0.2, and maximum tree depth = 15. GBM employed Bernoulli-distributed gradient boosting with 100 trees and interaction depth = 1. The core genes were defined as the intersection of genes selected by all seven algorithms.

### Core genes verification and analysis

The SSR2 gene was selected based on its differential expression between normal and tumor samples among the core genes and its AUC value as an independent diagnostic biomarker. The Survival package was applied to plot Kaplan–Meier curve with tumor samples from TCGA was divided into high risk group and low risk group using the best cutoff value. Then the tumor samples from TCGA was classified into high expression group and low expression group according to the median of SSR2 expression. A series of analysis was performed to evaluate the significance of SSR2, including immune cell correlation analysis, tumor microenvironment analysis, immune checkpoint analysis, GSEA, drug sensitivity analysis. Immune cell correlation analysis was performed with “spearman” method between the high and low expression groups using immune cell gene set from MSigDB, followed by calculating immune score with ssGSEA. The tumor microenvironment analysis was performed using estimate package. The gene list of immune checkpoints was downloaded from ImmPort database. The clusterProfiler package was applied to perform GSEA. The GDSC2 database was utilized in drug sensitivity analysis.

### NK cells reclustering

NK cells were selected by observing the expression levels of the SSR2 gene in different cell subsets, followed by normalization. Then 2000 highly variable genes were selected using FindVariableFeatures function. Subsequently, data scaling and PCA (30 principal components) was performed. Graph-based clustering was conducted using Louvain algorithm (FindNeighbors dims = 1:20; FindClusters resolution = 0.6). The AddModuleScore function was applied to evaluate the SSR2 expression in different NK cell subsets. NK cells were re-annotated as “SSR2 + NK” or “SSR2-NK”.

### Pseudotime analysis and differentiation potential analysis

Pseudotime analysis and differentiation potential analysis was employed to perform trajectory inference to model the developmental progression of NK cell subsets using monocle package [29, 30]. For trajectory Construction, genes with mean expression > 0.1 were selected for ordering, DDRTree algorithm was applied for dimensionality reduction (max components = 2). Pseudotime was calculated with root state set to cluster 5 (determined by minimum dispersion). Cellular potency was evaluated using CytoTRACE package. Differentiation potential analysis was based on gene counts per cell and expression diversity and default parameters was used: minimum 200 expressed genes per cell.

### Cell–cell communication analysis

Cell–cell communication analysis was performed to conduct systematic analysis of intercellular signaling networks using CellChat package [31, 32]. “CellChatDB.human receptor-ligand database” was selected and secreted signaling set was retained for analysis. This choice was made to focus on the pivotal role of cytokines and chemokines in orchestrating immune cell recruitment and immune evasion, which are central themes of the current study. Next, identifyOverExpressedGenes and identifyOverExpressedInteractions functions with default default parameters were applied to identify genes that significantly highly expressed within groups and potential receptor-ligand pairs. Followed by, the communication probability of each receptor-ligand pair between different cell subsets was calculated using computeCommunProb function.

### Clinical specimens

Tumor samples, along with corresponding adjacent peritumoral tissues, were obtained from patients who underwent liver resection at the First Affiliated Hospital of Anhui Medical University. Written informed consent was obtained from all participants. All procedures adhered to the Declaration of Helsinki. This study was approved by the Institutional Review Boards of The First Affiliated Hospital of Anhui Medical University. The IRB approval numbers and animal use protocol numbers are 2024468 and LLSC20241358 respectively.

### Cell culture

Human hepatocellular carcinoma cell lines (Hep3B, HepG2, Huh7, MHCC97H and HCCLM3) and the normal human liver cell line MIHA were used for in vitro validation of SSR2 expression. Cells were cultured in Dulbecco’s modified Eagle’s medium (DMEM) or RPMI-1640 (as appropriate for each cell line) supplemented with 10% fetal bovine serum (FBS) and 1% penicillin–streptomycin at 37 °C in a humidified incubator with 5% CO₂. Cells were routinely tested to exclude mycoplasma contamination and were harvested at ~ 70–80% confluence for protein extraction.

### Western blotting

Frozen paired tissues (P and T) were homogenized in freshly prepared radioimmunoprecipitation assay (RIPA) lysis buffer supplemented with phenylmethylsulphonyl fluoride (PMSF) at a 100:1 (v/v) ratio. Homogenates were incubated on ice and then centrifuged at 12,000 rpm for 30 min at 4 °C. The supernatants were collected, and protein concentrations were determined using a bicinchoninic acid (BCA) assay. Equal amounts of protein were mixed with protein loading buffer at a 4:1 (v/v) ratio and denatured by boiling at 100 °C for 10 min.

Proteins were separated by SDS–PAGE (PAGE Gel Fast Preparation Kit) and transferred onto polyvinylidene fluoride (PVDF) membranes. Membranes were blocked in 5% non-fat milk prepared in TBST (Tris-buffered saline with 0.1% Tween 20) for 1 h at room temperature, followed by incubation with primary antibodies against SSR2 and GAPDH at 4 °C overnight. After three washes with TBST, membranes were incubated with the corresponding HRP-conjugated secondary antibodies for 2 h at room temperature and washed three additional times with TBST. SSR2 protein levels were normalized to GAPDH for subsequent statistical analyses.

### RT-qPCR

Total RNA was extracted from frozen paired tissues (P and T) using TRIzol reagent according to the manufacturer’s instructions. RNA purity and concentration were assessed by spectrophotometry, and cDNA was synthesized using a reverse transcription kit following the manufacturer’s protocol. Quantitative PCR was performed using a SYBR Green Master Mix on a real-time PCR system under the following conditions: initial denaturation at 95 °C for 30 s, followed by 40 cycles of 95 °C for 5 s and 60 °C for 30 s, with a subsequent melt-curve analysis to verify amplification specificity. Each sample was analyzed in technical triplicates. The primer sequences (5′ → 3′) were as follows: SSR2 forward, CACTGCTGAACAGATACGCCGT; SSR2 reverse, GCCAAAGTCTTCTGGAGGGAAG; GAPDHforward, GTCTCCTCTGACTTCAACAGCG; GAPDHreverse, ACCACCCTGTTGCTGTAGCCAA. GAPDH was used as the internal reference gene. Relative SSR2 mRNA expression was calculated using the 2^−ΔΔCt^ method, where ΔCt = Ct(SSR2) − Ct(GAPDH), and paired adjacent tissues (P) served as the calibrator for normalization. For paired comparisons between tumor tissues (T) and the corresponding adjacent tissues (P), a two-tailed paired Student’s t-test was used, with P < 0.05 considered statistically significant.

## Results

### scRNA-seq analysis constructed cellular atlas and identified functional pathways

**Fig. 2:**
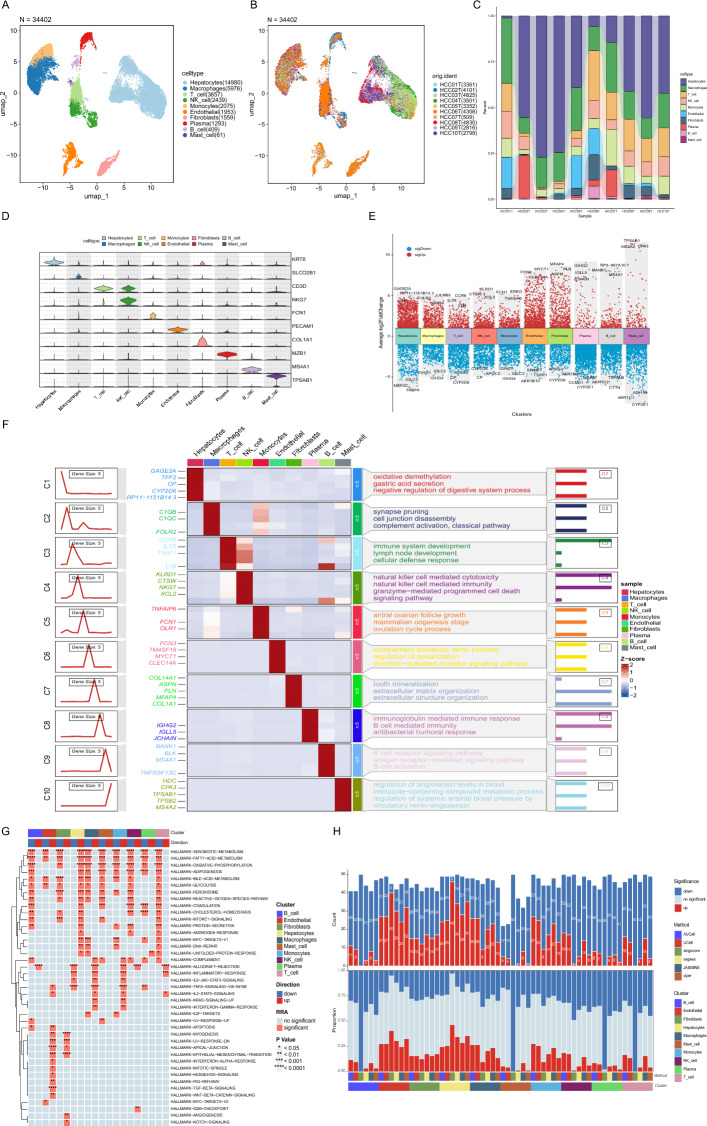
Analysis of single-cell transcriptome. **A** UMAP plot showing the distribution of 10 distinct cell types in liver tissues. **B** UMAP plot colored HCC sample source. **C** Bar chart revealing the cell proportion of each sample. **D** The marker genes of distinct cell types. **E** The differential expressed genes of each cell type. **F** GO over-representation analysis for BP. **G** Consensus hallmark pathways through GSEA. **H** The pathways enriched by different score calculation methods

To investigate the cellular composition and heterogeneity in the liver tissues from HCC patients, we performed clustering and annotation. A total of 34,402 cells were partitioned into 10 distinct subsets according their marker genes, including hepatocytes (KRT8), macrophages (SLCO2B1), T cell (CD3D), NK cell (NKG7), monocytes (FCN1), endothelial (PECAM1), fibroblasts (COL1A1), plasma (MZB1), B cell (MS4A1), mast cell (TPSAB1) in Fig. 2A and D. And we discovered that cell distribution varied among different patients, which may reveal the heterogeneity in tumor microenvironment in Fig. 2B and C. We identified differentially expressed genes (DEGs) across clusters in Fig. 2E. Through these differentially expressed genes, we identified the distinct pathways of each cell subsets in Fig. 2F. We got each cell subsets’ significant pathways using Hallmark GSEA in Fig. 2G and these pathways were consensus in different cell scoring method in Fig. 2F.

### Cytoplasmic translation pathway was selected with spatial transcriptome

**Fig. 3:**
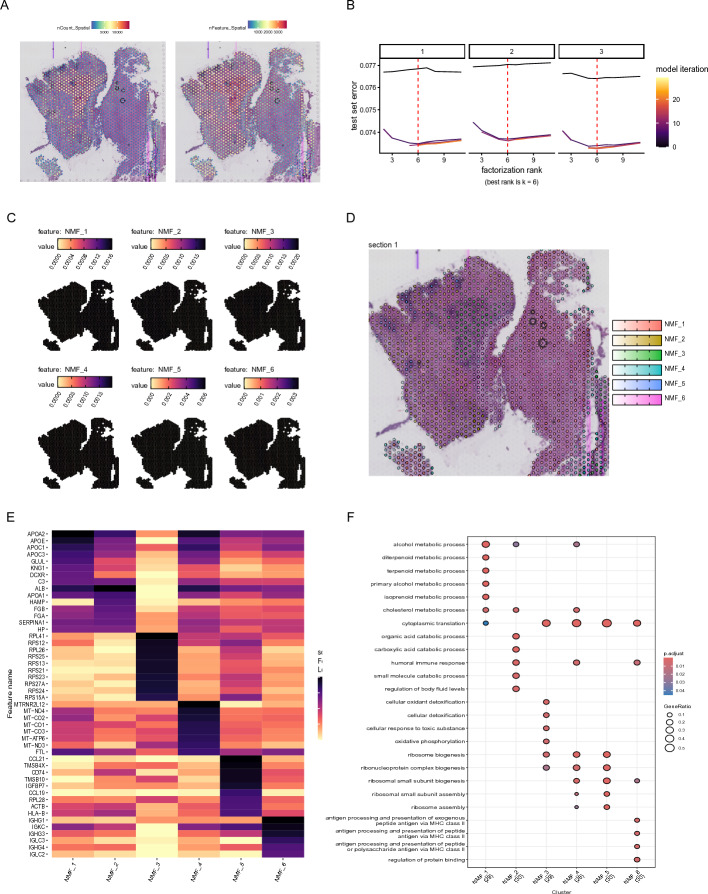
Analysis of spatial transcriptome. **A** The counts and features of every spot. **B** Best rank is k = 6 in NMF. **C**, **D** The distribution of 6 NMF components in spatial transcriptome. **E** Heatmap showing the top 10 high-loading genes of each NMF component. **F** GO over-representation analysis on top 30 high-loading genes of each NMF component

Through spatial transcriptome analysis, we obtained the total number of gene counts and the number of detected genes in different spatial position in Fig. 3A, which reflecting the level of transcriptional activity and gene diversity. According to test set error and model interaction, we chose k = 6 as the best factorization rank in Fig. 3B. Therefore, the spots of spatial transcriptome were divided into 6 NMF components in Fig. 3C and D. The heatmap showed the top 10 high-loading genes of each NMF component in Fig. 3E. By performing GO over-representation analysis on top 30 high-loading genes of each NMF component, we discovered that cytoplasmic translation pathway was enriched in most NMF components in Fig. 3F. NMF_1 and NMF_2 exhibited high expression of IgG-related genes, MHC-II molecules, and chemokines such as CCL19/CCL21, representing the tumor-associated immune infiltration regions. NMF_3 was associated with liver-specific structural proteins and represents a mixed tumor/hepatocyte transcriptional subtype. NMF_4 was significantly enriched in ribosome assembly, cytoplasmic translation and oxidative phosphorylation pathways, suggesting that it represents the high proliferation zone of tumor cells. NMF_5, featuring the genes HP, FGA, FGB, and SERPINA1, corresponds to an inflammatory/acute-phase response transcriptional signature. NMF_6 was rich in genes related to lipid metabolism (APOE, APOC1), reflecting the unique metabolic reprogramming characteristics of liver cancer. In conclusion, cytoplasmic translation path plays a significant functional role in tumor progression and may represent a potential therapeutic target.

### Essential gene modules were identified by hdWGCNA

**Fig. 4:**
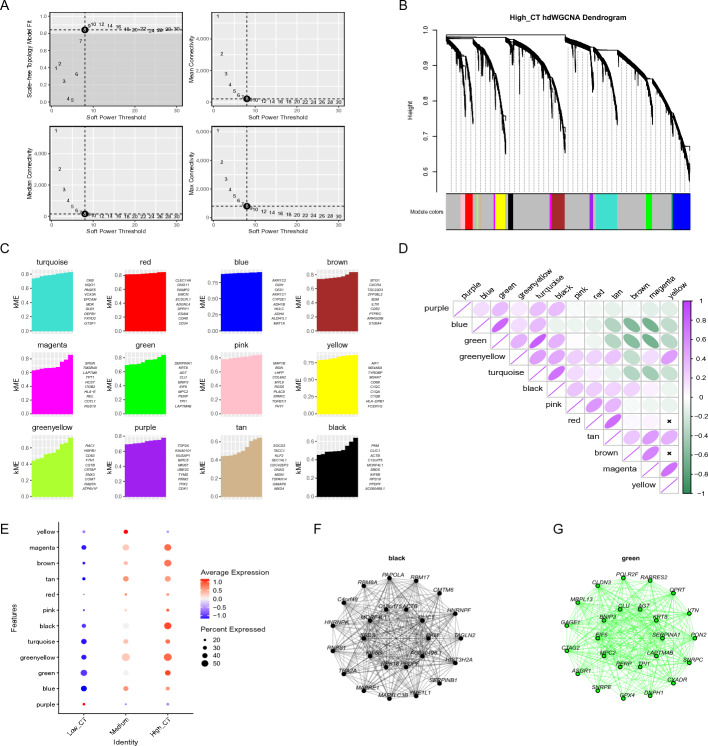
The hdWGCNA indicated the important gene modules. **A** Scale-free topology model fit degree, mean, median and max connectivity corresponding to different soft power thresholds. **B** The hdWGCNA dendrogram of 12 modules that have been identified. **C** The top genes in each module ranked by kME. **D** Analysis of the relationships between different modules. **E** The bubble plot showing black and green module had the greatest intergroup difference. **F**–**G** Representative gene networks from black and green module

We scored each cell in the single-cell transcriptome data based on the cytoplasmic translation gene set and obtained three groups: “High_CT” (top 1/3), “Low_CT” (bottom 1/3), and “Medium” (middle 1/3). Cells in the “High_CT” group showed a high level of cytoplasmic translation activity, and this activity corresponds to HCC. In order to further explore the gene modules that play a crucial role, we set the soft power threshold as 8 in Fig. 4A and obtained 12 gene modules (turquoise, red, blue, brown, magenta, green, pink, yellow, greenyellow, purple, tan and black) through hdWGCNA in Fig. 4B and C. Furthermore, we also evaluated the correlations of each module in Fig. 4D. Followed by, we analyzed the differences of distinct gene modules across the three groups (“High_CT”, “Low_CT”, “Medium”), discovering that black and green modules showed the greatest intergroup difference in Fig. 4E. Thereafter, the gene interaction networks corresponding to modules black and green was constructed in Fig. 4F. The modules black and green represented the co-express synergistically genes among cells with distinct activities in cytoplasmic translation pathway corresponding to HCC, which can help us to identify potential key HCC-related genes.

### Machine learning algorithms screened core genes

**Fig. 5:**
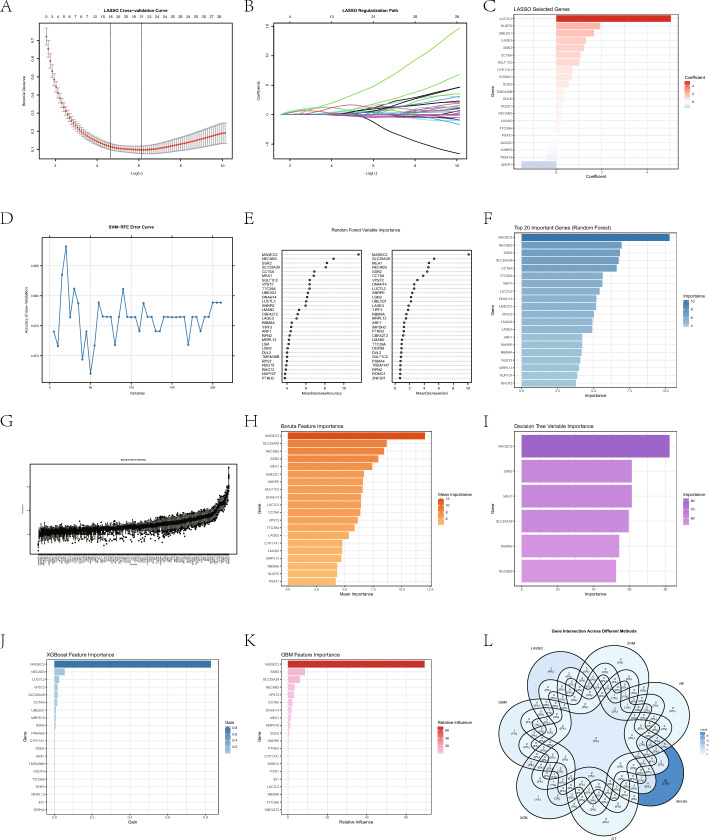
Machine learning screened core genes. **A**–**C** The results of LASSO algorithm for selection genes. **D** SVM-RFE algorithm for selection genes. **E**–**F** Results from the Random Forest algorithm. **G**, **H** The Boruta algorithm for selection genes were correlated to HCC. **I** The results of the Decision Tree algorithm. **J** The xGBoost algorithm selecting genes. **K** Gradient Boosting Machine algorithm screening genes related to HCC. **L** The Venn diagram illustrating 2 genes shared by seven machine learning algorithms

We used 7 machine learning algorithms to identify potential HCC biomarkers based on Bulk transcriptome data with module genes of black and green. The LASSO regression model selected 21 feature genes in Fig. 5A–C. The SVM-RFE method identified 20 candidate markers in Fig. 5D. The random forest (RF) model further confirmed 30 statistically significant variables with 500 trees in Fig. 5E, F. In parallel, the Boruta feature selection algorithm highlighted 64 genes in Fig. 5G, H. The decision tree (DT) algorithm identified 6 high-impact genes in Fig. 5I, while the XGBoost approach detected 20 key markers in Fig. 5J. Additionally, the GBM model selected 20 genes associated with HCC-related expression changes in Fig. 5K. The gene lists obtained from each of the 7 machine learning algorithms were summarized. Finally, by integrating the results across all models, we identified 2 optimal diagnostic biomarkers that were consistently selected by multiple algorithms: SSR2, NECAB3 in Fig. 5L.

### SSR2 was selected as an independent diagnostic biomarker

**Fig. 6:**
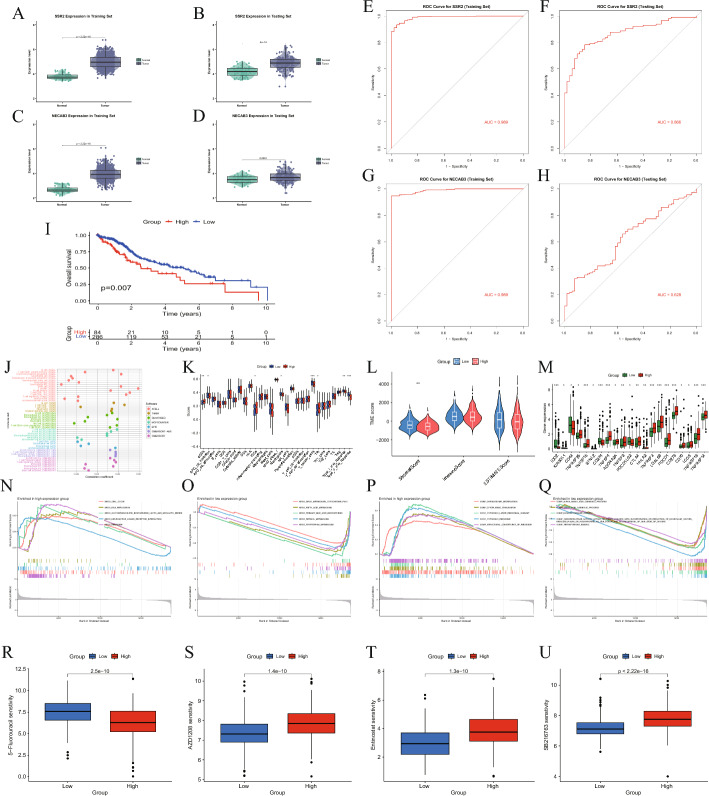
The SSR2 gene possessed excellent diagnosis and prognostic abilities. **A**, **B** The SSR2 expression difference in training set and testing set. **C**, **D** The NECAB3 expression difference in training set and testing set. **E**, **F** ROC curve for SSR2 in training set and testing set. **G**, **H** ROC curve for NECAB3 in training set and testing set. **I** KM curve for high and low risk groups stratified by SSR2. **J**, **K** Analysis of immune cell correlation between high and low SSR2 expression groups. **L** The results of tumor microenvironment analysis. **M** Results from immune checkpoint analysis. **N**–**Q** The KEGG and GO GSEA results of high and low SSR2 expression groups. **R**–**U** Analysis of drug sensitivity

The expression of SSR2 and NECAB3 showed significant differences in both the training set and the testing set constructed based on the bulk transcriptome data in Fig. 6A–D. We attempted to use SSR2 and NECAB3 as independent diagnostic biomarker respectively. The AUC results showed that SSR2 performed well in both the training set and the testing set, while NECAB3 performed poorly in the testing set in Fig. 6E–H. Therefore, we finally chose SSR2 as the independent diagnostic biomarker. Furthermore, Kaplan–Meier curve indicated that SSR2 is related to prognosis in Fig. 6I. Then we evaluated the differences in the tumor immune microenvironment between high and low SSR2 expression groups (Fig. 6J–M). We also analyzed the differences in functional pathways for high and low SSR2 expression groups in Fig. 6N–Q. Followed by, drug sensitivity analysis was conducted in Fig. 6R–U. Interestingly, we found that compared to the low SSR2 expression group, the high SSR2 expression group had lower 5-Fluorouracil sensitivity, while 5-Fluorouracil is a classic first-line drug used in clinical anti-tumor therapy. This seemed to indicate that its single-agent efficacy against HCC is low, and it relies on combination therapy. Therefore, the development of new drugs targeting HCC is necessary.

### Trajectory analysis and cell–cell communication analysis

**Fig. 7:**
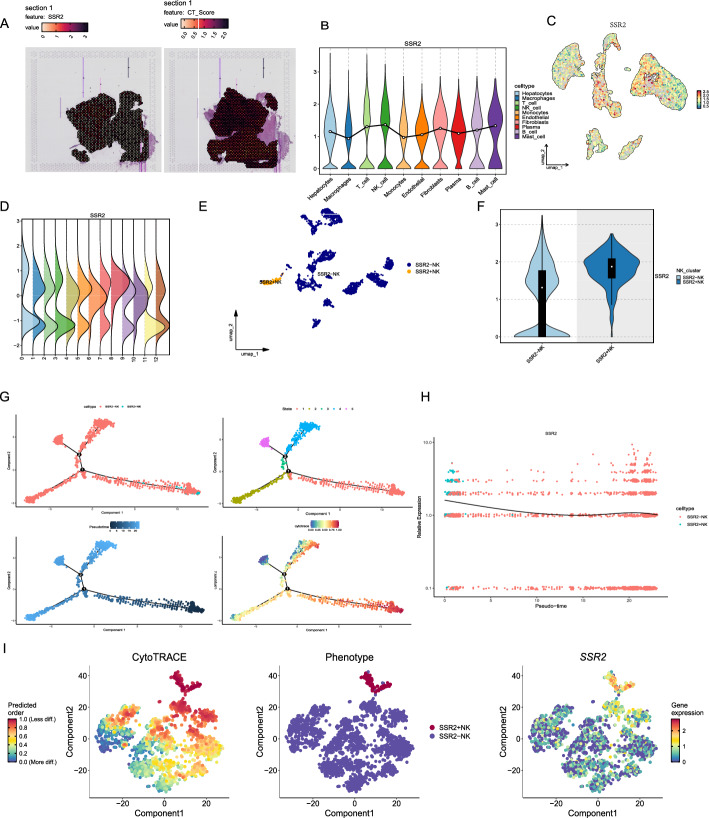
The expression level of SSR2 decreases as NK cells develop. **A** The expression distribution of SSR2 and the CT_score in spatial transcriptome. **B** Violin plot showing SSR2 had the highest average expression level in natural killer cells. **C** The expression distribution of SSR2 in single-cell transcriptome. **D**–**F** NK cells subset 8 was defined as “SSR2 + NK” due to the SSR2 expression level. **G**, **H** The results of pseudotime and differentiation potential analysis. **I** The t-SNE plot showing the differentiation potential, phenotype and SSR2 expression level of NK cells

**Fig. 8:**
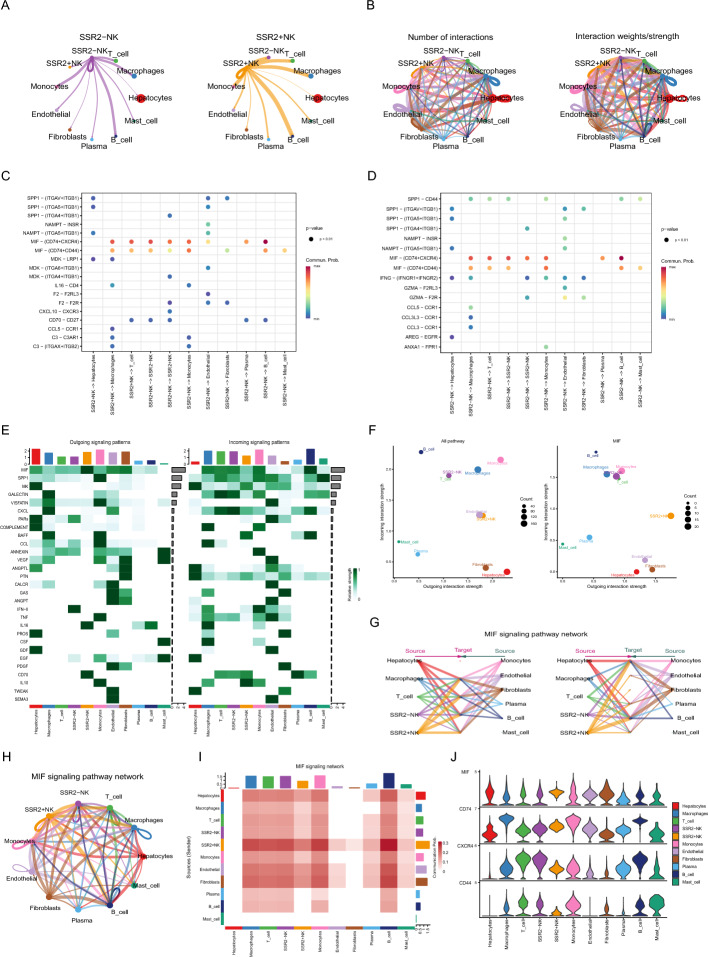
MIF-mediated signaling pathway play an important role. **A** The cell–cell communication results of “SSR2 + NK” cell and “SSR2-NK” cell. **B** Number of interaction and interaction weights/strength between distinct cell types. **C**, **D** Bubble plot showing MIF-mediated signaling pathway was significantly enriched in both “SSR2 + NK” and “SSR2-NK”. **E** The heatmap revealing outgoing signaling patterns and incoming signaling patterns. **F** The dot plot illustrating the difference between all pathway and MIF-mediated signaling pathway. **G**–**I** A detailed presentation of MIF-mediated signaling pathway. **J** Violin plot showing the expression level of MIF, CD74, CXCR4 and CD44 in different cell types

We separately evaluated the SSR2 expression level for spatial transcriptome and Single-cell transcriptome in Fig. 7A, C. Spatial mapping revealed a significant spatial congruence between SSR2 and the High_CT signature in Fig. 7A. Notably, while the majority of SSR2-high spots were precisely nested within High_CT regions, the High_CT signature occupied a broader spatial domain. This indicates that SSR2 specifically delineates a focal sub-population of highly cytotoxic NK cells within the broader activated immune landscape of the tumor microenvironment. To clarify its expression patterns across different cell types in Single-cell transcriptome, the RCTD algorithm was used. RCTD analysis allowed us to infer the major contributing cell types for each spot and to evaluate the relationship between SSR2 expression and the estimated abundance of different cell populations. We found that SSR2 had the highest average expression level in NK cells in Fig. 7B. The subset 8 of NK cells were re-annotated as “SSR2 + NK” and others as “SSR2-NK” in Fig. 7D, E. The SSR2 expression had a difference between “SSR2 + NK” and “SSR2-NK” in Fig. 7F. Pseudotime analysis and differentiation potential analysis indicated that the expression level of SSR2 decreases as NK cells develop in Fig. 7G-I. We conducted a further study on the intercellular communication of “SSR2 + NK” and “SSR2-NK” in Fig. 8A, B. The MIF-mediated signaling pathway was significantly enriched in both “SSR2 + NK” and “SSR2-NK” in Fig. 8C, D. Next, we investigated the MIF-mediated signaling pathway network among all cell types in Fig. 8E–J. Interestingly, our cell–cell communication analysis highlighted a functional bifurcation: while the “SSR2 + NK” subset exhibited the highest outgoing MIF signaling flux, the “SSR2-NK” subset was characterized by higher incoming signals with elevated expression of MIF receptors (CXCR4, CD44). This suggests that SSR2 might serve as a marker for MIF-producing effector NK cells, whereas the “SSR2-NK” population maintains higher sensitivity to MIF via receptor upregulation. Despite this receptor distribution, the integrated communication score remained dominant in the “SSR2 + NK” cluster, reinforcing its role as a key regulatory hub in the NK cell inflammatory response. Our findings identified that the MIF-mediated signaling pathway is essential for modulating the crosstalk between different cell types in the HCC tumor microenvironment.

### SSR2 is upregulated in hepatocellular carcinoma tissues

Compared with the paired adjacent tissues (P), tumor tissues (T) exhibited significantly increased SSR2 mRNA levels, as determined by RT–qPCR using GAPDH as an internal control in Supplementary Fig. 2D. Western blot analysis further revealed elevated SSR2 protein expression in T tissues, and quantitative densitometry normalized to GAPDH showed a concordant increase in Supplementary Fig. 2A, B. To provide in vitro evidence, SSR2 protein levels were assessed by western blot in HCC cell lines (Hep3B, HepG2, Huh7, MHCC97H and HCCLM3) and the normal liver cell line MIHA. SSR2 was consistently elevated across HCC cell lines relative to MIHA in Supplementary Fig. 2C, which corroborates our tissue-based findings. Together, these results suggest that SSR2 is upregulated in hepatocellular carcinoma tissues. To further strengthen the robustness of our findings, we validated SSR2 expression using independent external datasets, which consistently supported the upregulation of SSR2 in HCC.

## Discussion

HCC is among the most common and deadly cancers globally, marked by elevated recurrence rates and unfavorable clinical outcomes [33]. Increasing data demonstrates that the quantity of NK cells is significantly linked to patient survival; a recent meta-analysis revealed that elevated NK-cell levels are related with enhanced overall survival and disease-free survival [34]. Despite NK cells being a significant innate immune effector population in the liver, their functionality is often compromised within the HCC microenvironment, evidenced by functional exhaustion, diminished cytokine secretion, and dysregulation of activating and inhibitory receptors, thereby facilitating tumor immune evasion [35]. To clarify the processes of NK-cell dysfunction, we combined single-cell RNA sequencing (scRNA-seq) and bulk RNA-seq datasets, employing machine-learning techniques to discover pivotal genes closely linked to NK-cell functional states from extensive transcriptome data. This comprehensive analytical approach has demonstrated significant efficacy in other circumstances, including investigations that uncover regulatory elements that control mononuclear phagocyte infiltration in HCC [36]. Utilizing this methodology, we discerned an NK-cell subset inside the HCC microenvironment distinguished by significantly heightened expression of Signal sequence receptor subunit 2 (SSR2), indicating that SSR2 may serve as a defining molecular characteristic and potential regulatory element of this NK subpopulation. Our data indicate that SSR2 expression is higher in immature NK cell subsets and decreases upon maturation. Given the “exhausted” state of NK cells often observed in HCC, it is plausible that SSR2 plays a stage-specific role in maintaining NK cell homeostasis. Immature NK cells, characterized by high SSR2 levels, may represent a highly plastic population. In the tumor microenvironment, the sustained demand for protein synthesis and secretion might lead to ER stress; if SSR2-mediated protein translocation is insufficient or declines prematurely, it could accelerate the transition from a functional immature state to a terminal exhaustion state. Future studies are needed to determine if SSR2 levels directly correlate with exhaustion markers such as PD-1 or TIM-3 across different NK maturation stages. Analysis of cell–cell communication indicated that endothelial cells and fibroblasts are the primary producers of macrophage migration inhibitory factor (MIF) and presumably participate in MIF-receptor interactions with SSR2-high NK cells. These data suggest that the SSR2-MIF axis may be a pivotal mechanism underlying NK-cell suppression and immunological evasion in HCC, potentially providing novel therapeutic targets and strategic avenues for NK cell-based immunotherapies.

SSR2, also known as TRAP-β, is an endoplasmic reticulum (ER) membrane-associated protein involved in co-translational translocation of nascent polypeptides. SSR2 has recently been identified as significantly elevated in HCC. Numerous studies indicate that SSR2 overexpression correlates with increased proliferation and migration of hepatoma cells, together with unfavorable clinical outcomes [37–39]. Chen et al. revealed that increased SSR2 expression markedly enhances HCC cell proliferation and is inversely associated with patient prognosis [37]. Recent research reveals that SSR2 promotes HCC advancement via altering the Hippo system, suggesting that the SSR2/Hippo axis may serve as a potential treatment target [38]. SSR2 was recognized as a crucial gene particularly expressed, or significantly elevated, in particular NK-cell subsets, indicating its possible role in modulating NK-cell functioning. This discovery paves the way for further functional research to clarify the role of SSR2 in NK cells.

Meanwhile, MIF is a multifunctional inflammatory and immunoregulatory cytokine that plays a particularly important role within the tumor microenvironment. MIF derived from tumor stroma, such as cancer-associated fibroblasts, is considered a key regulator of tumor–immune cell communication [36]. Single-cell RNA sequencing (scRNA-seq) studies in HCC have revealed abundant ligand–receptor interactions involving the MIF–CD74 + CXCR4 receptor axis across multiple cell types, including immune cells such as NK cells [40, 41]. Structurally, MIF can initiate downstream signaling by binding to CD74 and forming a heteromeric receptor complex with co-receptors such as CXCR4 [42]. Furthermore, emerging evidence suggests that MIF can induce NK cells to secrete pro-inflammatory cytokines such as IFN-γ and modulate the expression of cytotoxic receptors (e.g., NKG2D and DNAM-1), thereby potentially enhancing NK-cell antitumor activity while, under conditions of excessive expression, paradoxically impairing their ability to recognize and eliminate tumor cells [43]. Previous studies in other tumor types also corroborates an immunoregulatory function of MIF in influencing NK-cell activity. Research on ovarian cancer indicates that MIF can downregulate NKG2D expression on NK cells, thereby reducing their cytotoxic effect [44]. While the HCC-specific MIF–NK axis has not been thoroughly delineated, our results align with these data, suggesting that MIF may serve as an underappreciated modulator of NK-cell suppression in HCC.

If future techniques effectively target SSR2 or impair MIF–NK communication—such as by suppressing MIF or obstructing its engagement with certain receptors—it may be feasible to augment the antitumor efficacy of NK cells in HCC. However, it is crucial to recognize the pleiotropic nature of MIF within the tumor ecosystem. As highlighted by Youness et al. [43], MIF exhibits a complex ‘dual role’: while it acts as a potent pro-inflammatory cytokine in early immune responses, it frequently pivots toward an immunosuppressive phenotype in advanced malignancies, orchestrating immune escape and tumor growth. In the context of HCC, the high expression of SSR2 may tilt the balance of the MIF axis toward this suppressive state, thereby impairing NK cell surveillance. These methodologies would offer novel conceptual pathways for the advancement of more efficacious NK cell–based immunotherapies, encompassing tailored NK-cell products and combinatorial methods with immune checkpoint inhibitors.

Certainly, our research possesses multiple limitations. While our bioinformatics investigations outlined a potential SSR2–MIF interaction network, these findings are yet inferential. Functional validation, encompassing in vitro co-culture assays, SSR2 knockout or overexpression investigations, and receptor-blocking studies, is critically required to elucidate the molecular relevance of this axis. Furthermore, the single-cell datasets employed were sourced from public repositories, where tumor stage, sample processing, and patient demographics may differ significantly, thus influencing the generalizability of our results. Moreover, while we identified endothelial cells and fibroblasts as primary sources of MIF, spatial molecular methodologies, including immunohistochemistry and spatial transcriptomics, are necessary to confirm the in situ spatial distribution and cellular localization of MIF within HCC tissues.

## Supplementary Information

Supplementary Material 1. Supplementary Figure 1: RCTD deconvolution results of spatial transcriptomics data

Supplementary Material 2. Supplementary Figure 2: SSR2 expression in hepatocellular carcinoma. (A,B) Western blotting results. (C) SSR2 protein expression in liver cell lines..(D) RT–qPCR results. (E-F) Transcriptomic expression analyses.

Supplementary Material 3.

## Data Availability

The data used in this study can be accessed in online databases, including scRNA-seq from GEO (Gene Expression Omnibus) database (Accession ID: GSE149614, https://www.ncbi.nlm.nih.gov/geo/query/acc.cgi?acc=GSE149614), spatial RNA-seq from GEO database (Accession ID: GSM6177612, https://www.ncbi.nlm.nih.gov/geo/query/acc.cgi?acc=GSM6177612), Bulk transcriptome data from TCGA (The Cancer Genome Atlas, https://portal.gdc.cancer.gov) database and GEO database (Accession ID: GSE76427, https://www.ncbi.nlm.nih.gov/geo/query/acc.cgi?acc=GSE76427). The detailed information can be found in the article. If you have any further questions, please contact us.
