# Supplementary figures and images for: Exploration of the roles of SSR2 in hepatocellular carcinogenesis based on single-cell transcriptomics and spatial transcriptomics
Source: Discov Oncol. 2026 Apr 14;17:799. doi: 10.1007/s12672-026-05017-w (PMC13201726; doi:10.1007/s12672-026-05017-w)

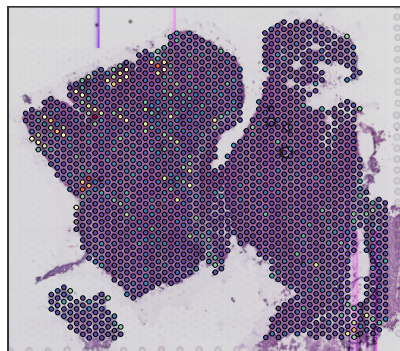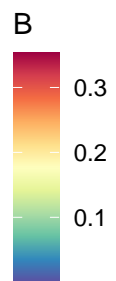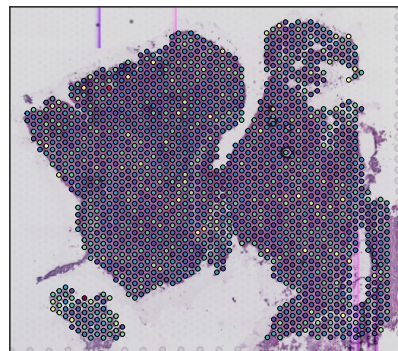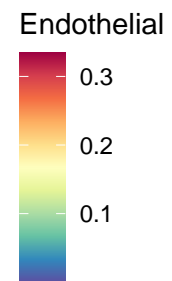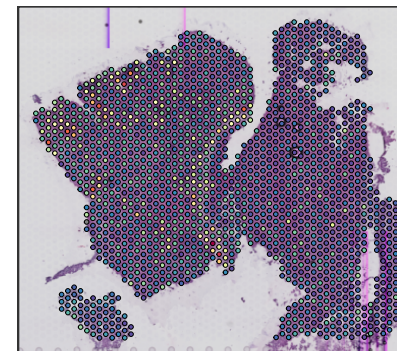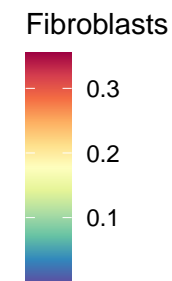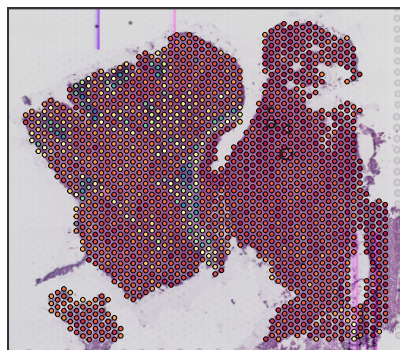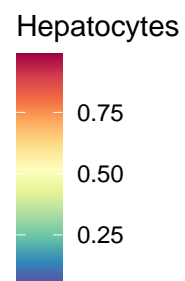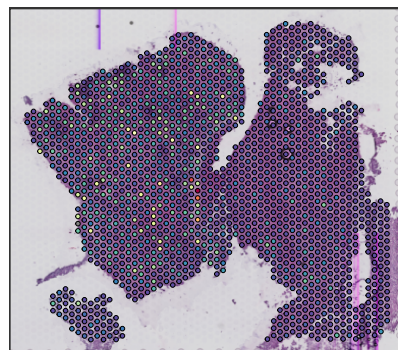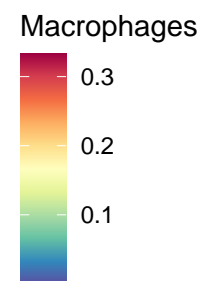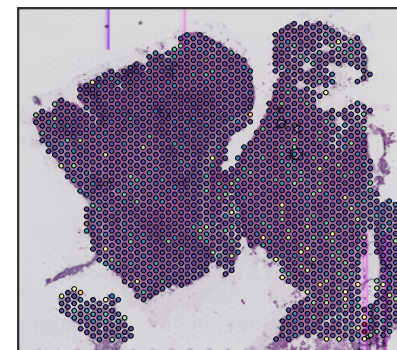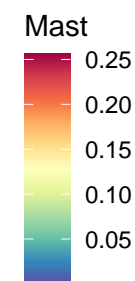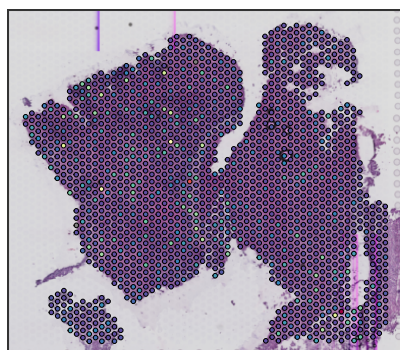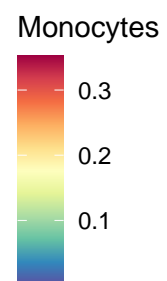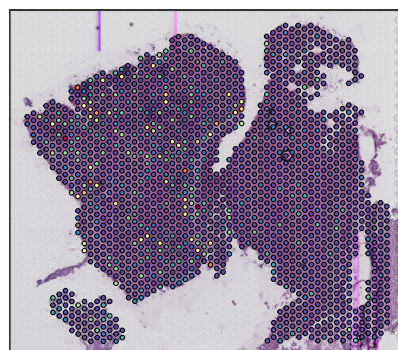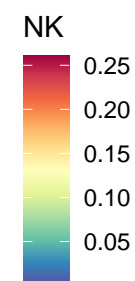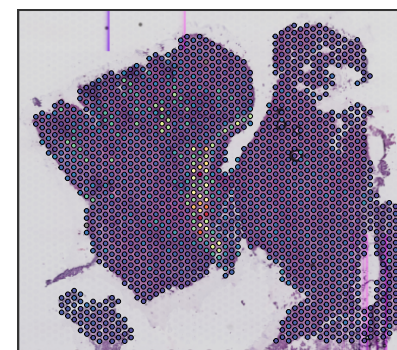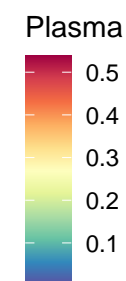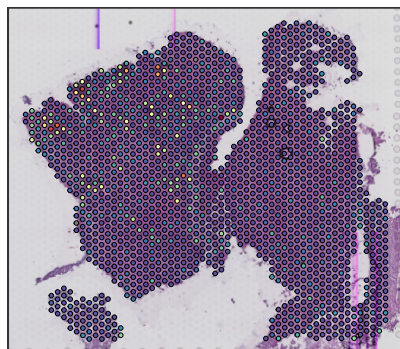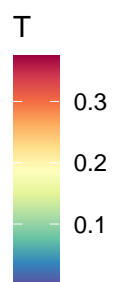

Supplement: Supplementary file 1 — Supplementary Material 1. Supplementary Figure 1: RCTD deconvolution results of spatial transcriptomics data [file 12672_2026_5017_MOESM1_ESM.pdf]

A

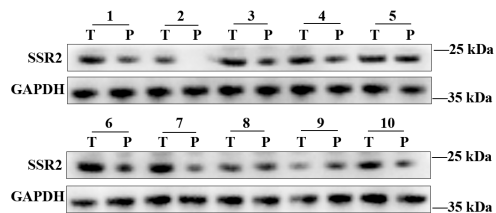

B

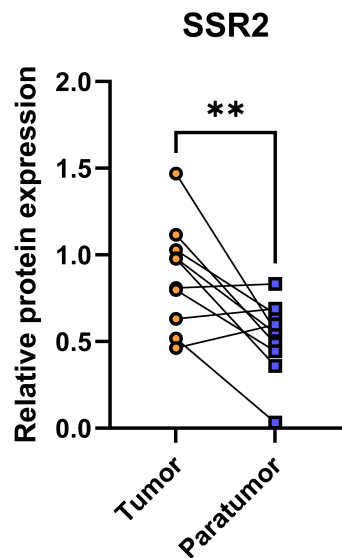

C

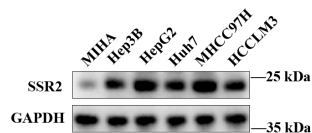

D

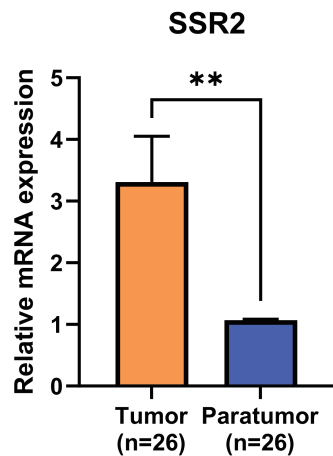

E

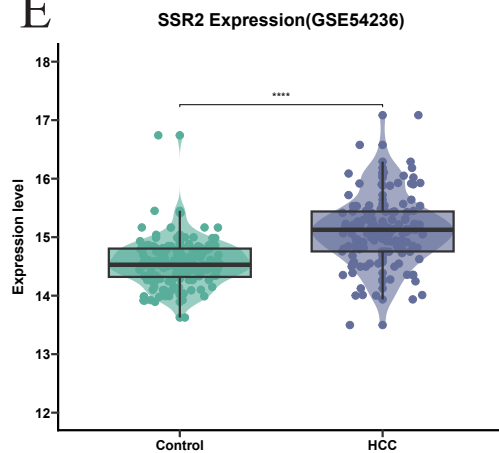

F

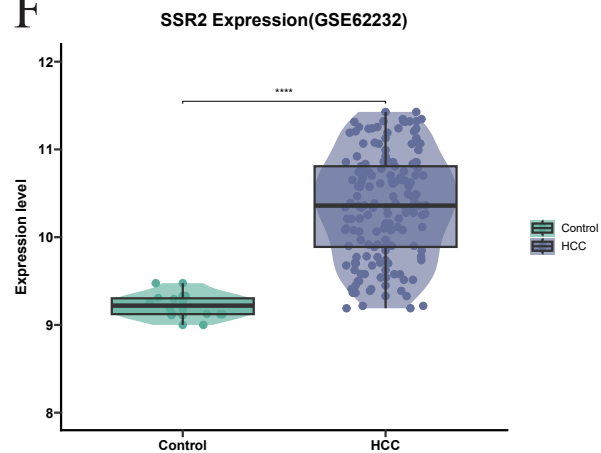

Supplement: Supplementary file 2 — Supplementary Material 2. Supplementary Figure 2: SSR2 expression in hepatocellular carcinoma. (A,B) Western blotting results. (C) SSR2 protein expression in liver cell lines..(D) RT–qPCR results. (E-F) Transcriptomic expression analyses. [file 12672_2026_5017_MOESM2_ESM.pdf]

# WB uncropped original image

Figure S2 A

SSR2

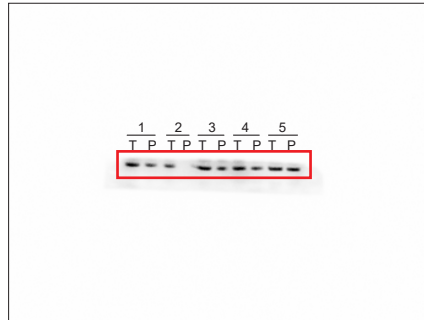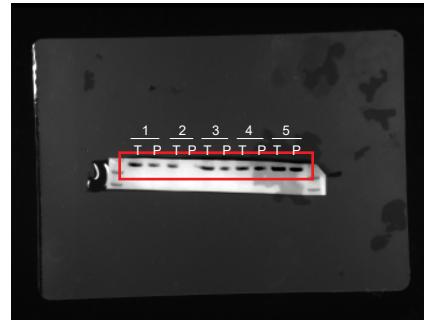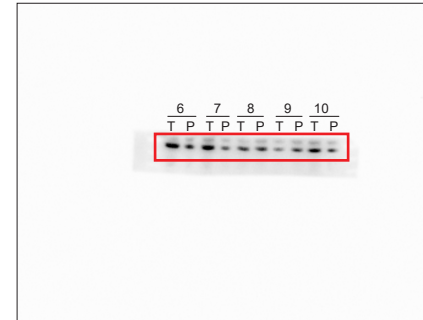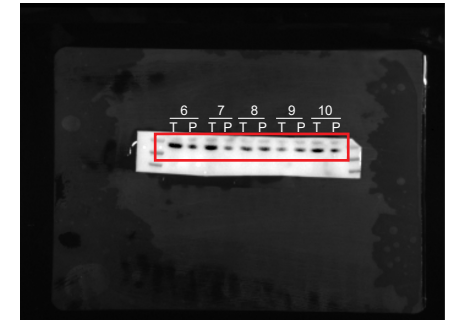

GAPDH

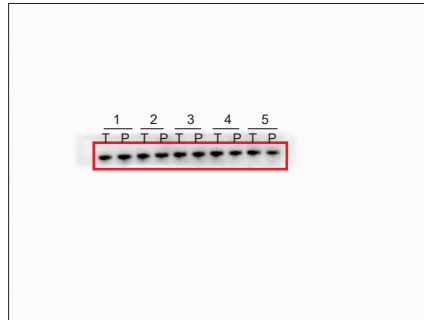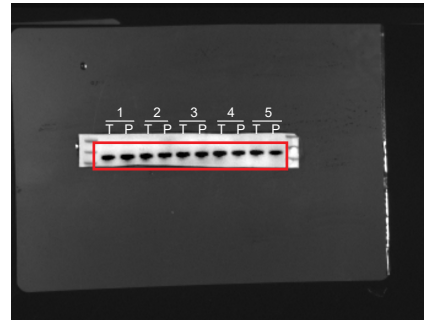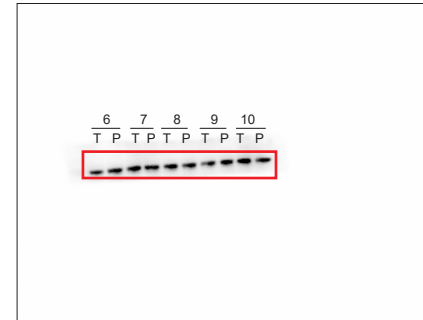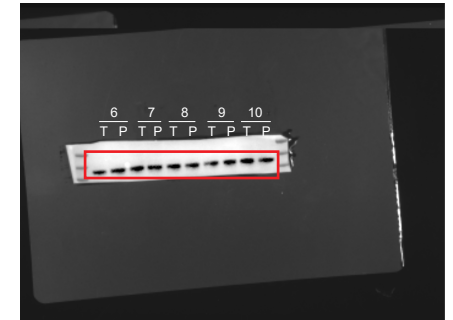

Figure S2 C

SSR2

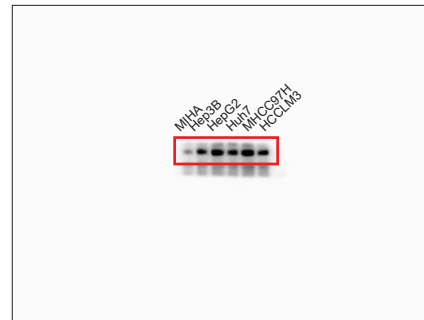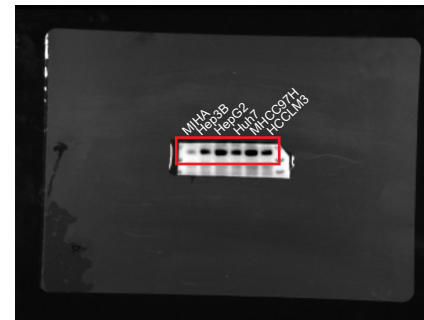

GAPDH

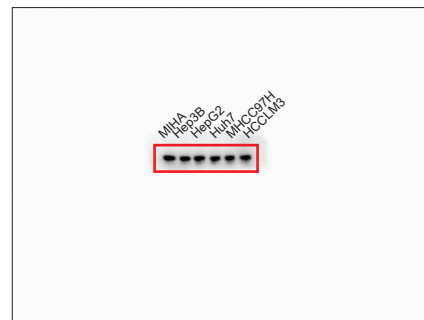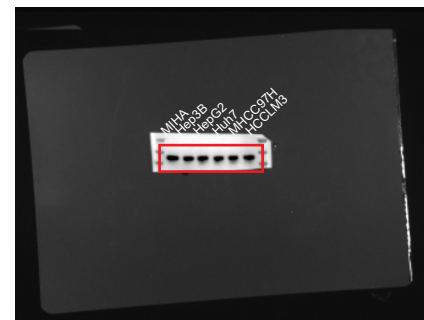

Supplement: Supplementary file 3 — Supplementary Material 3. [file 12672_2026_5017_MOESM3_ESM.pdf]
